# From the operating room to the cloud: the evolving continuum from live surgical broadcasting to remote tele-surgery

**DOI:** 10.1007/s11701-026-03962-w

**Published:** 2026-10-13

**Authors:** Priyank Yadav, Husam H. Balkhy, Mohan S. Gundeti

**Affiliations:** 1 Department of Surgery, Section of Urology, The University of Chicago Medicine & Biological Sciences Division, Chicago, IL USA; 2 Department of Surgery, Section of Cardiac and Thoracic Surgery, The University of Chicago Medicine & Biological Sciences Division, Chicago, IL USA

**Keywords:** Tele-surgery, Live surgical broadcast, Tele-proctoring, Robotic surgery, Digital medicine, Surgical education, 5G, Health equity, Remote surgery

## Abstract

In the last four decades, the transmission of a live surgical operation to a remote audience has evolved from closed-circuit educational broadcast into a fully remote, robot-assisted surgery performed across different continents. This review maps that journey systematically, defining a six-rung Digital Surgical Spectrum — from passive live broadcast to artificial-intelligence-augmented autonomous surgery — grounding each stage in published clinical evidence across multiple surgical domains. We demonstrate that each rung of this spectrum is no longer theoretical. Live broadcasts have reached audiences across multiple countries. Tele-proctoring was validated at scale during the COVID-19 pandemic, and tele-mentoring has been shown in systematic review to produce a safety and efficacy profile broadly comparable to on-site supervision. True remote tele-surgery has been accomplished over distances exceeding 8,000 km with latencies well within established procedure specific safety thresholds. We also present a case timeline and an Institutional Readiness Framework specifying the technical, regulatory, and other prerequisites for each rung, organized visually as a Digital Surgical Spectrum. Importantly, nuances such as the performance paradox of observed surgery, consent frameworks, cybersecurity risk, regulatory fragmentation, and the threat of entrenching rather than correcting global surgical inequity are examined. As the field advances, the growth of tele-surgical technologies will depend on international regulatory harmonization and equity-centered deployment, supported by prospective multi-specialty outcome registries.

## Introduction

In May 2026, a cardiac surgeon in Georgetown, Guyana, performed a robotic left internal mammary artery takedown on a patient in Indore, India, separated by nearly 20,000 km — the longest-distance robotic telesurgery yet reported [1]. The reported network latency was 290–300 milliseconds, and the four-hour, fifty-minute procedure was completed without adverse events [1].

This event did not represent a sudden breakthrough, but rather the culmination of nearly three-decade evolution in digitally mediated surgery, whose true origin point is Lindbergh Operation – the September 2001 transatlantic telerobotic cholecystectomy in which a surgeon in New York operated on a patient in Strasbourg, France [2]. Since then, technological capabilities, connectivity, and surgical workflows have advanced incrementally, first through live intraoperative video feeds and interactive proctoring, and more recently through 5G and dedicated-fiber networks capable of supporting true remote instrument control at scale. Between those cases in 2001 and 2026 lies a continuous spectrum of technologically mediated surgical practice — one that has progressed rung by rung, with each stage building on the infrastructure, evidence base, and operational lessons of its predecessors.

However, the pace of technological innovation now outstrips the development of governance. There is no widely accepted taxonomy for digitally mediated surgical interventions, and current regulatory and ethical frameworks do not adequately address their clinical, legal, and societal implications. Transparent, systematic discussion of the tensions introduced by these technologies is limited, particularly around safety, liability, data governance, and equity.

In this review, we synthesize multi-specialty data to evaluate safety, efficacy, and equity across this evolving continuum, highlighting both the strength of the existing evidence and the key gaps that remain. We introduce a conceptual model — the Digital Surgical Spectrum — to organize current and emerging practices and present a milestone timeline and an Institutional Readiness Framework to structure implementation considerations. Finally, we examine the critical nuances that will determine whether these technologies realize their potential to expand access and quality or instead reproduce and amplify existing inequities.

Although this is a narrative rather than a systematic review, relevant literature was identified through PubMed and Google Scholar searches through July 2026 using combinations of the terms “telesurgery,” “remote surgery,” “telementoring,” “teleproctoring,” “live surgical broadcast,” “robotic surgery,” and “5G,” supplemented by hand-searching of reference lists and professional-society statements; landmark cases were cross-checked against primary reports where available.

### The digital surgical spectrum: a unifying framework

The literature on remotely mediated surgery is characterized by inconsistent and imprecise terminology. Terms such as “tele-proctoring,” “tele-mentoring,” and “tele-remote surgery” are often used interchangeably, which risks obscuring materially different technical requirements, risk profiles, and ethical implications [3].

**Fig. 1:**
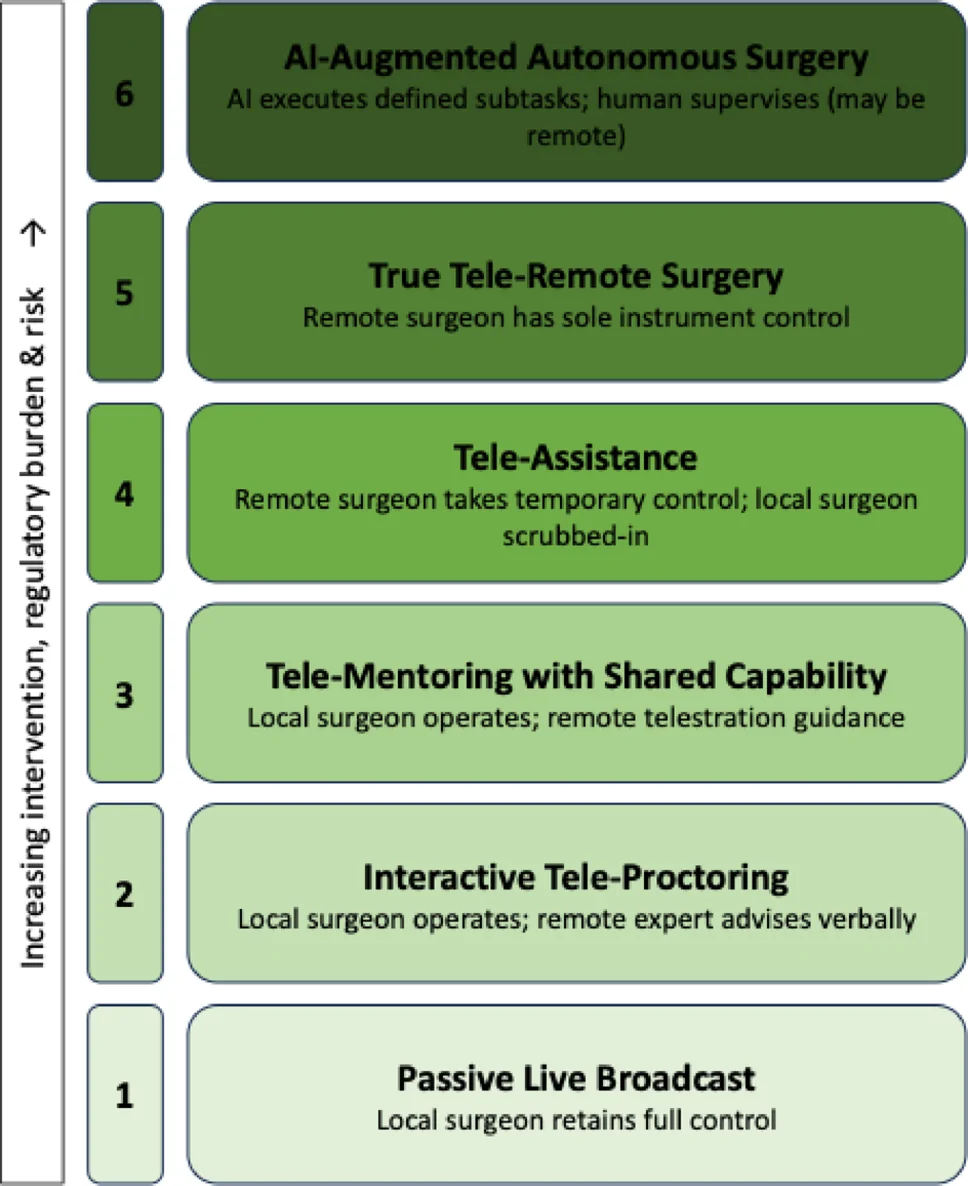
The Digital Surgical Spectrum. Rungs are cumulative in demand but not mutually exclusive

To address this, we propose a unifying framework: the Digital Surgical Spectrum. This spectrum is conceptualized as a six-rung ladder, in which each upward step represents a qualitative change in the nature of the surgical intervention, rather than a merely quantitative increase in technological complexity. Figure 1 provides a visual representation of this framework, summarizing the control model, technical threshold, and consent requirement that characterize each rung.

Each rung is associated with progressively demanding prerequisites across five domains: technical infrastructure, regulatory compliance, patient consent, training and competence, and failsafe planning. The rungs are not mutually exclusive; institutions may operate at multiple levels simultaneously. For example, a center may function at Rung 3 for robotic urological procedures while using Rung 1 live broadcasts for basic surgical education at the same time. The framework is intended as a map of capability, not a rigid or prescriptive linear pathway. While the six rungs describe increasing degrees of remote human involvement, they simultaneously map onto a second, orthogonal dimension: the degree of machine autonomy. These two axes are conceptually distinct. Rungs 1 through 5 hold machine autonomy essentially constant (the human retains full moment-to-moment control of every instrument movement) while varying who exercises that control and from what distance, escalating from passive observer (Rung 1) to sole remote operator (Rung 5). For rung 6, the change is no longer human distance but machine autonomy, as the system itself executes defined subtasks while the human transitions from operator to supervisor. A center advancing up the ladder is therefore not simply adopting better technology; it is progressively transferring accountability away from the bedside.

### The six rungs: evidence and achievements

#### Rung 1 — passive live broadcast

Live surgical broadcasting is a mature practice, but its risks remain understudied. Specialty broadcast platforms and society-run channels stream operative procedures to audiences spanning multiple countries, and several meetings routinely incorporate live case demonstrations as a component of continuing professional development. Surgeons report high educational value from observing expert technique in the context of real anatomical variation and intraoperative decision-making.

However, the evidence base for live broadcasting is predominantly observational. Published data do not consistently show worse patient outcomes with live surgery broadcasts, but the evidence is limited, heterogeneous, and some studies raise safety concerns in selected procedures and settings. Survey data consistently demonstrate high perceived educational benefit, yet robust prospective studies linking live broadcasts to measurable skill acquisition or improved patient outcomes are rare. Important negative nuances are often overlooked. First, performance psychology suggests that observation can alter expert behavior through phenomena such as the Hawthorne effect and “choking under pressure.” No surgical study has systematically evaluated whether surgeons performing live broadcasts make different intraoperative choices or convert to open surgery at different rates. Second, case selection is typically biased toward technically straightforward or visually striking procedures, which may distort perceptions of typical case complexity and risk. Third, the shift from closed conference halls to open internet platforms means that broad “educational use” consent forms are increasingly inadequate for archived videos that may be viewed by large, global audiences. Standardized, patient-informed broadcast-consent frameworks are lacking.

In response, some surgical societies have moved to prohibit live surgical broadcasts outright. The Society of Thoracic Surgeons guidelines explicitly state that surgeons “should not participate in live surgery broadcasts to the public or lay audiences”; the American College of Surgeons and the American College of Obstetricians and Gynaecologists have moved in a similar direction [4, 5]. In contrast, other bodies continue to permit live surgery under stringent conditions rather than banning it outright: the Royal College of Surgeons of England permits live broadcasts provided educational benefit clearly outweighs risk and robust consent and governance arrangements are in place [6]. A cross-specialty systematic review of published society guidelines likewise concluded that most surgical societies allow live broadcast only with rigorous safeguards, rather than prohibiting it entirely [5].

#### Rung 2 — interactive tele-proctoring

Adding bidirectional communication transforms passive viewing into active guidance while preserving local control of the instruments. Tele-proctoring involves a remote expert providing real-time verbal guidance and assessment to an operating surgeon. During the COVID-19 pandemic, travel restrictions and the need to maintain surgical training and credentialing accelerated the adoption of such models [7]. The available evidence is largely retrospective but generally reassuring.

Experimental studies suggest that latencies up to approximately 400–500 ms can be acceptable for verbal and visual guidance alone, though performance and surgeon comfort deteriorate as delay increases [8]. Much stricter latency requirements — typically in the ≤ 200–300 ms range — become critical when remote surgeons assume direct control of instruments in true telesurgery rather than tele-proctoring roles [8]. Regulators and professional bodies have begun to address remote surgical proctoring, although policy remains uneven. Contemporary implementation checklists describe tele-proctoring as an emerging modality that should employ secure, HIPAA-compliant video platforms with appropriate business-associate agreements, high-definition video, and reliable audio, rather than consumer videoconferencing tools configured without adequate data-protection safeguards [9].

#### Rung 3 — tele-mentoring with shared capability

Tele-mentoring extends beyond remote credentialing to support longitudinal development of surgical capability. It uses high-definition audiovisual links and, in many systems, telestration of the operative field to facilitate real-time guidance at a distance. In robot-assisted surgery, such platforms have been integrated into dual-console environments, enabling remote experts to provide stepwise instruction without demonstrable compromise of perioperative safety in early reports.

Hinata et al. described a telementoring system for robot-assisted radical prostatectomy that provided three-dimensional visualization, an overview camera, and annotation tools, comparing outcomes for the initial 30 patients operated on with telementoring against those operated on with direct mentoring; the two groups showed no significant differences in operating time, complication rate, early continence, or positive-margin rate [10]. In general surgery, Schlachta et al. implemented a longitudinal mentoring and telementoring program for laparoscopic colorectal resections between a university center and a community hospital, demonstrating that sustained remote support can facilitate safe technology transfer with outcomes consistent with expected benchmarks [11].

Synthesizing 66 studies across multiple surgical specialties, Erridge and colleagues concluded that telementoring appears to have a broadly similar safety and efficacy profile to on-site mentoring, although the evidence base is heterogeneous and dominated by lower-level study designs [12]. A complementary systematic review restricted to studies directly comparing telementoring with on-site mentoring reported similar complication rates and operative times, while noting that the level of evidence supporting telementoring as an educational intervention remains limited [13]. Other reviews highlight promise for telementoring in settings with limited access to subspecialist expertise, including low-resource and remote environments, while also underscoring the need for structured communication training and explicit attention to human factors in remote mentor–mentee interactions [14, 15].

#### Rung 4 — tele-assistance

Tele-assistance, in which a remote surgeon assumes temporary active operative control with a scrubbed on-site surgeon immediately available, marks the transition from purely advisory roles to direct (even if intermittent) remote operative participation. Moschovas and colleagues demonstrated this control-handoff model directly: surgeons in Orlando, Florida, and Shanghai, China, approximately 13,000 km apart, took turns controlling a single robotic platform during a two-day porcine study, transferring instrument control between locations multiple times per case, including during critical operative steps, while maintaining a median round-trip latency of 139 ms [16]. The authors described this as exploring telesurgery’s “teleproctoring potential”, with a remote expert stepping in to assist during complex or challenging moments rather than running the entire case. This is fundamentally different than Rung 5 where the entire control is transferred for the duration of the procedure. As an animal-model study, it demonstrates the control-transfer mechanism rather than clinical outcomes.

At this rung, ethical and regulatory questions become more acute, because the remote surgeon is directly manipulating instruments rather than providing advice alone. Licensure, jurisdiction, liability, and accountability are variably addressed across health systems, and there is currently no widely adopted framework that specifically governs cross-border operative tele-assistance. This regulatory fragmentation poses a substantial barrier to broader implementation beyond carefully controlled programs.

#### Rung 5 — true tele-remote surgery

The landmark trajectory of true tele-remote surgery begins with Lindbergh Operation in 2001 (Table 1), when Marescaux and colleagues performed a transatlantic telerobotic cholecystectomy, with a surgeon in New York operating on a patient in Strasbourg, France (approximately 6,200 km away) over a dedicated asynchronous transfer mode (ATM) fiber network with a reported round-trip latency of approximately 155 ms^2^. Their detailed reporting of network architecture, latency management, image quality, and intraoperative communication provided a reference framework for subsequent telesurgical experiments. However, it did not become so popular as the technology was costly and required a significant investment in infrastructure to guarantee effective signal transmission over long distances.

Anvari and colleagues established a more sustained model for remote surgical service: surgeons at St Joseph’s Healthcare in Hamilton, Ontario, provided laparoscopic procedures to patients at North Bay General Hospital, approximately 400 km away, via a dedicated IP-VPN broadband link [17]. In their initial series of 21 laparoscopic procedures — 13 fundoplications, 3 sigmoid resections, 2 right hemicolectomies, 1 anterior resection, and 2 inguinal hernia repairs — the authors reported no serious intraoperative complications and no conversions to open surgery, with hospital stays comparable to equivalent procedures performed locally, demonstrating that telesurgery could be implemented as a sustained clinical service rather than a one-off experiment [17].

Early 5G protocols were limited to advanced telementoring. For instance, Lacy and colleagues demonstrated 5G-assisted laparoscopic mentoring within Barcelona (~ 4 km) and Shanghai (~ 6 km), achieving manageable latencies but stopping short of controlling the physical instruments remotely [18]. However, true tele-remote surgery has since breached the clinical barrier. Recent international consensus highlights a major shift in true tele-remote surgery, moving from pre-clinical testing to expansive human clinical cohorts utilizing multi-port, single-port, and open or immersive consoles across general surgery, urology, cardiac surgery and gynecology [19]. Operating over 5G networks paired with dedicated lines or standard internet architectures, these systems have successfully logged hundreds of live human procedures. Reported latency depends heavily on distance and connection type: within 200 km, average delay is approximately 24–41 ms, rising to about 52 ms at 1,000–2,000 km, and at 5,000 km reaching 73 ms over a dedicated line versus 159 ms over 5G/LAN [19]. To safely breach these boundaries, pre-clinical animal models and simulators rigorously verified signal transmission over extreme intercontinental distances ranging from 13,000 km to over 20,000 km, demonstrating that robust communication architectures can support remote manipulation even on a global scale [16, 19–21].

A major unresolved technical limitation at this rung is the absence of haptic feedback. Current commercially available robotic systems provide no true force reflection to the surgeon, and although research platforms have demonstrated transmission of force information over networks, these systems have not been adopted for routine clinical tele-remote surgery. Cybersecurity constitutes a second critical challenge: robotic systems connected to hospital networks represent potential targets for intrusion, manipulation, or denial-of-service attacks. A recent survey and failure-mode analysis found that fewer than half of clinical respondents were familiar with robotic-assisted-surgery cybersecurity, and that only about a quarter of clinical respondents were aware of their own organization’s cybersecurity policy, underscoring the need for robust cybersecurity frameworks to mitigate associated patient-safety risks [22].

#### Rung 6 — AI-augmented autonomous surgery

This rung encompasses systems in which artificial intelligence performs defined surgical subtasks autonomously, under human supervision that may itself be remote. It is the only rung where the evidence base remains predominantly preclinical, but progress is real and substantive. In 2016, Shademan and colleagues reported that the Smart Tissue Autonomous Robot (STAR) successfully performed supervised autonomous intestinal anastomosis in a porcine model, producing suture lines that were more consistent than those achieved by manual human surgery [23]. Subsequent iterations of autonomous systems have demonstrated that they can reliably execute subtasks such as tissue grasping, needle driving, and knot tying, primarily in ex vivo animal-tissue models, with selected in vivo demonstrations.

The anticipated trajectory is incremental automation of well-defined surgical subtasks, introduced stepwise as evidence accumulates. As this occurs, the role of the remote human surgeon is likely to evolve from direct tele-operator to supervisory controller, with responsibility for task allocation, oversight, and intervention when autonomous performance deviates from expected parameters. Analogous models already exist in aviation and industrial robotics, where human operators supervise semi-autonomous systems, intervening at critical junctures rather than executing every action directly.

This transition has far-reaching implications. It will necessitate reconfiguration of surgical training curricula to include competencies in supervising and troubleshooting autonomous systems, revision of hospital credentialing processes to account for hybrid human–machine workflows, and re-examination of legal and ethical accountability frameworks to clarify responsibility when AI-driven actions contribute to adverse events.

**Table 1:** Landmark documented tele-remote surgical procedures across specialties

Year	Specialty	Procedure	Distance	Latency	Key finding
2001	General surgery	Cholecystectomy (Operation Lindbergh) [2]	New York → Strasbourg, France (~ 6,200 km)	~ 155 ms (asynchronous transfer mode fibre)	First intercontinental tele-robotic operation; demonstrated feasibility of transatlantic surgery over a dedicated ATM network.
2003	General surgery	Laparoscopic fundoplication, colorectal resection, hernia repair [17]	Hamilton → North Bay, Canada (~ 400 km)	Not formally quantified; no conversions or latency-attributed complications in 21 cases	First clinically implemented, sustained remote surgical service; established a rural-access model for tele-assistance.
2019	General / colorectal	Laparoscopic telementored resection [18]	Barcelona, Spain (~ 4 km) and Barcelona → Shanghai (~ 6 km)	146 ms and 202 ms respectively (5G)	First 5G-assisted telementored surgeries; demonstrated feasibility of real-time telestration guidance over commercial 5G.
2022	Urology	Robot-assisted urological procedure (renal cyst deroofing) [19]	Xinjiang (Kezhou) ↔ Jiangsu, China (~ 5,000 km)	> 310 ms (5G)	Longest-range 5G tele-robotic urological surgery reported at the time; demonstrated feasibility of ultra-long-distance 5G connectivity.
2024	Urology	Robotic radical prostatectomy [24]	Beijing ↔ Rome, Italy (~ 8,000 km)	135 ms (5G/**fibre**)	First intercontinental live-broadcast tele-remote surgery; no adverse events reported.
2025	Urology	Robotic radical prostatectomy [25]	Florida → Angola (~ 11,000 km)	146 ms (fibre)	Longest-range tele-robotic urological surgery reported at the time
2025	Cardiac Surgery	robotic atrial septal defect (ASD) closure [26]	Strasbourg, France → Indore, India (~ 6,400 km)	Not formally reported (described as “imperceptible”)	First intercontinental robotic cardiac telesurgery; no adverse events reported.
2026	Cardiac Surgery	left internal mammary artery (LIMA) takedown [1]	Guyana → Indore, India (~ 20,000 km)	290–300 ms (fibre)	Longest-distance robotic telesurgery ever performed

### Critical nuances: what the technology does not resolve

Across all rungs of the Digital Surgical Spectrum, five tensions recur that technical innovation alone cannot resolve, and that the field has not yet addressed adequately.

### Access versus accountability

The most compelling argument for tele-remote surgery is its potential to deliver expert surgical care to populations in remote or resource-limited settings where specialist expertise is unavailable locally. The Lancet Commission on Global Surgery estimated that five billion people lack access to safe, affordable surgery when they need it, with the greatest deficits in sub-Saharan Africa, South Asia, and parts of Latin America [27]. Tele-surgery, in principle, could address a meaningful fraction of this unmet need. In practice, however, the accountability structures required to make this safe — clear liability frameworks for cross-border operative complications, malpractice jurisdiction when surgeon and patient are subject to different legal systems, and insurance coverage for remote surgical events — are largely absent in current law and policy in most jurisdictions. Without these structures, the most likely outcome of tele-surgical expansion is not equitable global access, but concentration of the technology in well-resourced settings where institutional liability risk can be managed.

### Fidelity versus feasibility

The safest tele-surgical experience — ultra-low latency, high-definition stereoscopic video, redundant connectivity, encrypted transmission — requires infrastructure that exists in very few places globally. The communities that stand to benefit most from tele-remote surgery are precisely those least likely to have access to the 5G networks, reliable power supplies, and trained local technical support that safe operation requires. Deploying tele-surgery over inadequate infrastructure creates patient-safety risks — high latency, intermittent connectivity, degraded video — that may outweigh the access benefit. The field therefore requires a research agenda that prioritizes development of robust, lower-bandwidth tele-surgical systems capable of safe operation in real-world, resource-limited network environments, rather than continuing to focus disproportionately on optimal-condition landmark demonstrations. Several of the most recent landmark cases catalogued in Table 1 and elsewhere are documented only in trade press or manufacturer announcements [1, 21, 24, 26] rather than peer-reviewed reports and should be interpreted with corresponding caution; they are included here for completeness, not as evidence of equivalent methodological rigor to the peer-reviewed series.

### Training versus dependence

Tele-mentoring has substantial potential to accelerate surgical training, but the relationship between remote support and the development of genuine surgical independence warrants careful scrutiny. Analogous concerns have been documented in aviation, where autopilot systems that routinely intervene to correct errors have been associated with degradation of manual flying skills over time. Tele-mentoring programs should incorporate validated competency frameworks that specify the conditions under which remote support is withdrawn and should benchmark independent performance before a surgeon is considered fully credentialed.

### Innovation versus regulation

Robotic systems capable of tele-remote operation are commercially available in multiple jurisdictions; the regulatory frameworks for approving and governing their use over networks and across borders are not. The result is fragmentation: what is permissible in one country may be illegal in another; what is approved as research in one setting may be deployed clinically in another where the distinction is ambiguous. No major regulator has yet defined a clearance pathway specifically for cross-border teleoperation of a surgical robot, leaving even landmark intercontinental cases to proceed without a settled approval framework. Compounding this is an unresolved question of data governance, which is inseparable from the regulatory problem. Tele-surgery continuously transmits identifiable clinical data across networks that routinely span legal jurisdictions. There are no adopted standard specifies – (a) whose law governs that data when the surgeon, patient, and server reside in three different countries, (b) who owns and may retain the operative recording, or (c) how valid consent for cross-border data transfer should be obtained. The same recording can fall simultaneously under different regimes such as HIPAA and the GDPR, with materially different obligations attaching to it depending on where each party sits. National regulatory bodies should therefore treat tele-surgery governance as a priority, drawing on established frameworks for remote medical-device operation in cardiology and radiology as starting points, and should specify jurisdiction of record, data-stewardship and retention rules, and cross-border consent requirements as core components rather than afterthoughts.

### Broadcast ethics versus patient dignity

As operative procedures migrate from closed rooms to global digital platforms, the ethical standards surrounding patient consent for broadcast require urgent revision. Broad consent for “educational use” of operative footage — once adequate for a closed conference transmission — is not sufficient for public internet streaming to an unlimited audience with indefinite archival. Patients have a right to understand specifically who will observe, in what context, whether footage will be recorded, and how their anonymity will be protected if a complication occurs and the footage becomes subject to public or medicolegal scrutiny. This is particularly important for vulnerable populations — children, patients with cognitive impairment, and individuals in health systems where the practical ability to refuse consent is constrained by implicit power asymmetries.

### Future directions

Several converging developments will define the next phase of the Digital Surgical Spectrum. The deployment of low-earth-orbit satellite internet constellations offering global, low-latency connectivity — including in geographically remote areas currently unreachable by 5G infrastructure — is likely to extend the geographic reach of tele-remote surgery substantially within the next five years. Practical haptic-feedback systems for tele-surgery, currently at the advanced prototype stage, will change the ethical calculus of remote operation of complex procedures by restoring to the remote surgeon the tactile information that is currently absent. Augmented-reality overlays — in which a remote expert annotates the operative field seen by the local surgeon, marking safe dissection planes or highlighting critical structures in real time — are already in clinical use for certain applications and represent a near-term enhancement of tele-mentoring capability that does not require additional network infrastructure beyond that already used for video transmission.

At the system level, the most important future development is the establishment of prospective, multi-specialty outcome registries for tele-surgical procedures. Individual platforms are already accumulating substantial single-system experience — one commercial tele-robotic system has reportedly supported over one hundred human tele-surgical cases across general surgery, urology, and gynaecology [19] — but this experience remains fragmented across manufacturers, countries, and reporting standards. International surgical societies, working with regulators, should prioritize the development of a standardized minimum dataset for tele-surgical procedures that enables cross-institutional and cross-national outcome comparison. Table 2 presents the Institutional Readiness Framework proposed in this review as a practical planning tool for institutions and a potential template for accreditation-standards development.

**Table 2:** Institutional Readiness Framework for the Digital Surgical Spectrum

Domain	Rung 1: Broadcast	Rung 2: Tele-proctoring	Rung 3: Tele-mentoring	Rung 4: Tele-assistance	Rung 5: Tele-remote surgery
Technical	HD camera; streaming platform; standard internet	Secure bidirectional video; <500 ms latency	Dual-console robot or shared-view platform; <300 ms	Haptic-capable platform; redundant connectivity; <200 ms	5G/dedicated fibre; <150–155 ms; cybersecurity hardening; failsafe
Regulatory	Institutional broadcast-consent policy	Remote proctoring recognized by surgical board	Jurisdictional licensure; shared liability framework	Cross-border surgical license; dual-site anesthesia coverage	Full device approval (e.g., FDA/CE); cross-border liability and insurance
Patient consent	Broadcast-specific informed consent	Notification of remote-proctor identity	Consent for remote-control element	Explicit consent for remote operative intervention	Full consent including contingency plan if connectivity fails
Training	Surgeon + audiovisual technical staff	Remote-proctor credentialing	Dual-console training; communication protocol	Simulation-validated remote-intervention skills	Tele-surgery certification; simulation benchmarking required
Failsafe	-	Defined escalation path to on-site supervision	On-site supervisor available throughout	On-site surgeon scrubbed and ready	On-site surgeon fully prepared; automatic connectivity monitoring with abort protocol

## Conclusion

The Digital Surgical Spectrum is not a vision of the future: it is the present, distributed unevenly across institutions, specialties, and health systems. Live surgical broadcasts reach global audiences daily. Tele-proctoring is recognized by major surgical boards. Tele-mentoring has demonstrated a broadly comparable safety and efficacy profile to on-site mentoring across multiple specialties in systematic review. True tele-remote surgery has been performed over intercontinental distances in multiple landmark cases and advancements are being made towards AI-augmented autonomous surgery. What is absent is not the technology, but the governance framework — the shared taxonomy, the prospective evidence base, the regulatory standards, and the equity-centered deployment strategies — that would allow this technology to fulfil its transformative potential responsibly and for the populations who need it most.

## Data Availability

No datasets were generated or analysed during the current study.
